# Rifampin Use in Staphylococcal Prosthetic Joint Infections

**DOI:** 10.7759/cureus.96244

**Published:** 2025-11-06

**Authors:** Kunjan Shah, Christopher B Baladad, Alaina S Ritter, Kathryn DeSear

**Affiliations:** 1 Department of Pharmacy, University of Florida Health Shands Hospital, Gainesville, USA; 2 Department of Infectious Diseases and Global Medicine, College of Medicine, University of Florida, Gainesville, USA

**Keywords:** adverse events, prosthetic joint infection (pji), rifampin, staphylococcal pji, treatment duration

## Abstract

Background

Prosthetic joint infection (PJI) remains one of the most serious complications of prosthetic joint implantation. For patients with PJI managed with debridement, antibiotics, and implant retention (DAIR) surgical approach, two to six weeks of targeted intravenous antibiotics plus rifampin is typically recommended. Subsequently, rifampin plus a second oral antibiotic is given for a total of three versus six months for hip and knee arthroplasty infections, respectively. The goal of our study was to evaluate whether adjunctive rifampin affects treatment outcomes in patients with staphylococcal PJI.

Methods

We performed a retrospective study including patients aged > 18 with a diagnosis of prosthetic hip or knee infection due to staphylococcal species from January 2016 to December 2021. Patients were excluded if they did not receive appropriate anti-staphylococcal antibiotics, required resection arthroplasty or amputation, received a two-stage exchange as the initial surgical treatment, or were treated at another institution. The primary endpoint was treatment failure. Secondary endpoints included implant removal, long-term suppressive oral antibiotic utilization, hepatotoxicity, early rifampin discontinuation, and PJI-related mortality within one year.

Results

A total of 34 patients were included in the study, with 16 patients in the rifampin group (RIF) and 18 patients in the non-rifampin group (non-RIF). Most patients underwent DAIR for PJI management in both groups: 13/16 (81.3%) in RIF vs. 11/18 (61.1%) in non-RIF. The most common staphylococcalspecies causing infection was methicillin-susceptible *Staphylococcus aureus* (MSSA) in both groups. Treatment failure occurred in 2/16 (12.5%) of RIF patients vs. 9/18 (50%) of non-RIF patients (P = 0.030). The median duration of RIF therapy was 42 days, with 2/16 (12.5%) patients discontinuing rifampin early due to drug intolerance.

Conclusions

Recommendations for rifampin use in PJI vary in the literature. Despite its limitations, this study shows that at our institution, there was a statistically significant benefit conferred by rifampin usage in PJI treatment.

## Introduction

Advancements in joint replacement procedures have significantly improved mobility, independence, and limb functioning for patients with severe joint pain [1]. As a result, the number of joint replacements in the United States has steadily increased over the past decade, with approximately one million total hip and knee replacement procedures performed each year [2,3]. As the number of joint replacements rises, however, the occurrence of potentially serious complications such as prosthetic joint infection (PJI) has also increased. Criteria for defining PJI include the presence of a sinus tract communicating with the prosthesis, acute inflammation identified in periprosthetic tissue obtained intraoperatively, the presence of purulence around the prosthesis without any other known cause, and two or more joint cultures yielding the same organism or one culture with a pathogenic organism, as well as the clinical intuition of the evaluating provider [1]. The cumulative incidence of PJI over the lifespan of a prosthesis ranges from 1% to 2%, depending on the type of prosthesis and the occurrence of subsequent surgeries [1,3-8].

Current Infectious Diseases Society of America (IDSA) guidelines for the medical treatment of staphylococcal PJI recommend a two to six-week course of targeted intravenous (IV) antibiotics combined with rifampin 300-450 mg taken orally twice daily for patients undergoing a debridement, antibiotics, and implant retention (DAIR) surgical management strategy. The induction treatment is followed by rifampin plus a pathogen-specific oral antibiotic for a total of three months in the setting of a total hip arthroplasty (THA) PJI and six months in the setting of a total knee arthroplasty (TKA) PJI. Rifampin should be given in combination with other antimicrobials due to its low barrier to resistance. While rifampin has activity against staphylococcal biofilms that form on metal implants, the role of rifampin use as monotherapy or combination therapy as part of extended chronic suppression remains controversial, however. The decision to pursue chronic suppression with rifampin is therefore often made on a case-by-case basis, taking into consideration an individual patient’s needs and circumstances [1].

While IDSA guidelines recommend the use of rifampin in the initial treatment for PJI, some studies have raised questions regarding its efficacy. For example, a multi-center, randomized controlled trial conducted in Norway found no statistically significant difference in the rate of early postoperative and acute hematogenous staphylococcal PJI between the non-rifampin monotherapy (72%) and rifampin combination therapy (74%) groups. It is important to note that this study did not include PJI caused by methicillin-resistant *Staphylococcus aureus* (MRSA), which is a common pathogen for PJI [9]. Additionally, the literature often focuses primarily on *S. aureus* or staphylococcal species altogether as opposed to coagulase-negative staphylococci (CoNS) alone [9-10]. Given the lack of consensus on this topic, our study aimed to evaluate whether adjunctive rifampin affected treatment outcomes for PJI caused by staphylococcal species at our institution.

## Materials and methods

This was a retrospective cohort study conducted at the University of Florida Health System (UF Health), Gainesville, Florida, United States, a 1,162-bed academic medical center. The study was approved by the Institutional Review Board of the University of Florida and determined to be exempt.

Data were collected from the electronic medical record and included patients aged 18 through 89 who were diagnosed with prosthetic hip or prosthetic knee infection between January 2016 and December 2021. Patients were included if the infection was caused by any rifampin-susceptible *Staphylococcus* spp. with culture results including those obtained either directly from the prosthesis intraoperatively, from synovial fluid culture, through polymerase chain reaction (PCR), or through next-generation sequencing. Patients were excluded if cultures showed CoNS that was felt to be a contaminant and therefore not treated, if the patient required permanent resection arthroplasty or amputation as the initial surgical procedure, if the patient was initially treated at an outside institution, if there was no reported or available culture data, if the surgical treatment was a two-stage exchange, or if the PJI was polymicrobial with non-*Staphylococcus* spp. present.

Data were collected for two groups: (i) Non-rifampin group (non-RIF), in which PJI was treated with pathogen-directed or empiric antimicrobial therapy, and (ii) Rifampin group (RIF), in which PJI was treated with the addition of rifampin therapy. Organism identification and antimicrobial susceptibility results were obtained using VITEK 2 AST-GP72 cards (bioMérieux SA, Marcy-l'Étoile, France). Antimicrobial susceptibility testing (AST) results were interpreted according to the Clinical Laboratory Standards Institute (CLSI) M100 breakpoints from 2022 [11]. Antimicrobial dosing was renally adjusted by the treating clinicians per the established renal dosing protocol, and institutional pharmacists assisted with therapeutic drug monitoring based on antibiotic and patient-specific factors. Of note, all patients on vancomycin at our institution automatically receive pharmacy consults.

The primary endpoint of this study was treatment failure, defined as the persistence or relapse of the staphylococcal infection, reinfection with a new microorganism, or the need for any further surgical procedure related to the infection within one year after the initial procedure. The secondary endpoints included the incidence of implant removal, the need for long-term suppressive oral antibiotics, any instances of hepatotoxicity, early discontinuation of rifampin due to adverse drug events (ADEs), and mortality related to PJI within one year. Implant removal was defined as the removal of any part of an implant, excluding modular exchanges. The need for long-term suppressive oral antibiotics was confirmed by the retention of the implant or the inability to clear the infection due to the presence of a retained prosthesis. Resistance to rifampin or initial drug therapy was defined as either resistant susceptibility results from VITEK or persistent culture positivity despite being on therapy, determined to be susceptible by VITEK. Hepatotoxicity was defined as an increase in liver function tests (aspartate transaminase and alanine transaminase) from baseline by three times the upper limit of normal (ULN) based on institutionally designated reference ranges. Halting rifampin earlier due to ADEs was defined as the occurrence of any ADE directly attributed to rifampin or resulting from an interaction with rifampin. PJI-related mortality was defined as death caused by factors associated with PJI or its causative pathogen, such as sepsis, bacteremia, surgical complications, bleeding, or joint failure leading to falls.

Descriptive statistics were utilized to summarize the baseline demographics and results. For parametric data, continuous variables were presented as mean and standard deviation (SD), while non-parametric data were described using median and interquartile range (IQR). Categorical variables were reported as counts and percentages. Inferential statistics were conducted using Student’s t-tests, as all continuous variables were normally distributed. Categorical variables were analyzed using either the Chi-squared test or Fisher's exact test, when appropriate. Multivariate regression analysis was not performed due to the small sample size. Statistical significance was defined as a p-value less than 0.05. All statistical analyses were conducted using JMP Pro software v15.0 (SAS Institute Inc., Cary, North Carolina, United States).

## Results

**Figure 1:**
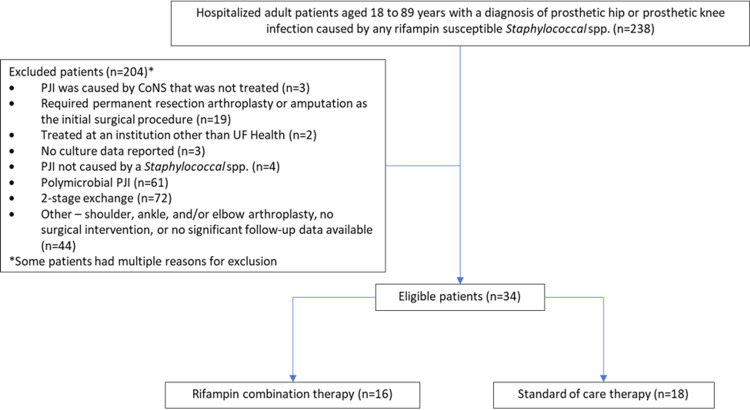
Inclusion/exclusion flowchart PJI: prosthetic joint infection; CoNS: coagulase-negative staphylococci

A total of 238 patients were screened for inclusion in the study. Of these, 204 patients were excluded, leaving 34 patients who met the inclusion criteria. Of the excluded patients, four patients did not have *Staphylococcus* spp. as the causative agent of the original PJI, 19 patients required permanent resection arthroplasty or amputation as the initial surgical procedure, two patients were treated at a non-UF Health institution, three patients had no culture data reported, 61 patients had polymicrobial PJI, three patients had CoNS that were not treated, 72 patients underwent two-stage exchanges, and 44 patients were excluded for other reasons (Figure 1).

Most patients included in the study were of Caucasian descent, comprising 93.8% of patients in the RIF group and 72.2% of patients in the non-RIF group. Of note, 44% of patients in the non-RIF group had diabetes compared to only 6.3% of patients in the RIF group (P = 0.019). A greater percentage of patients in the RIF group (81.3%) underwent DAIR surgical treatment compared to the non-RIF group (61.1%). Osteoarthritis was the primary indication for prosthesis in both groups, although the RIF group had a higher percentage of patients with this condition compared to the non-RIF group (87.5% vs. 66.7%). Non-continuous variables are presented in Table 1, and continuous variables are presented in Table 2.

**Table 1:** Demographics (non-continuous) *All chi-square values are from the Pearson chi-squared test. Only race had a degree of freedom (df) of 2, whereas all other variables had a df of 1. RIF: rifampin

Variables	RIF Combination (n=16)	Non-RIF (n=18)	Chi-square value*	P-value
Sex (male), n (%)	7 (43.8)	12 (66.7)	1.804	0.300
Race			3.370	0.297
Caucasian/White, n (%)	15 (93.8)	13 (72.2)		
African American/Black, n (%)	0	3 (16.7)		
Other, n (%)	1 (6.3)	2 (11.1)		
Comorbidities				
Diabetes, n (%)	1 (6.3)	8 (44.4)	6.349	0.019
Hypertension, n (%)	12 (75)	14 (77.8)	0.036	1
Cirrhosis, n (%)	1 (6.3)	0	1.159	0.471
Disease requiring systemic anticoagulation, n (%)	1 (6.3)	2 (11.1)	0.249	1
Chronic kidney disease, n (%)	2 (12.5)	1 (5.6)	0.508	0.591
Rheumatoid arthritis, n (%)	2 (12.5)	1 (5.6)	0.508	0.591
Immunosuppressed, n (%)	0	3 (16.7)	2.925	0.230
Prosthesis indication				
Arthritis				
Osteoarthritis, n (%)	14 (87.5)	12 (66.7)	2.043	0.233
Rheumatoid arthritis, n (%)	2 (12.5)	1 (5.6)	0.508	0.591
Connective tissue disorder, n (%)	1 (6.3)	2 (11.1)	0.249	1
Trauma, n (%)	1 (6.3)	1 (5.6)	0.007	1
Other, n (%)	0	2 (11.1)	1.889	0.487
Prosthetic Joint Type			0.360	0.682
Total Hip Arthroplasty, n (%)	4 (25)	3 (16.7)		
Total Knee Arthroplasty, n (%)	12 (75)	15 (83.3)		
Surgical Treatment Strategy			1.655	0.270
DAIR, n (%)	13 (81.3)	11 (61.1)		
1-stage Exchange, n (%)	3 (18.8)	7 (38.9)		

**Table 2:** Demographics (continuous) *All t-values were obtained from a pooled t-test. RIF: rifampin; ALT: alanine aminotransferase; AST: aspartate aminotransferase

Variables	RIF Combination (n=16)	Non-RIF (n=18)	t-value*	Degrees of freedom (df)	P-value
Age (years), median (IQR)	67.9 (57.7-75.5)	65.2 (59.5-71.2)	-1.143	32	0.131
Weight (kg), median (IQR)	90 (78.3-104.8)	89 (67-133)	0.282	32	0.610
Height (in), mean ± SD	66.6 ± 4.3	68.2 ± 4.9	0.999	32	0.837
C-reactive protein at start of treatment (mg/L), median (IQR)	114.3 (42.2-300)	156.6 (75.9-257.8)	-0.207	28	0.419
Erythrocyte sedimentation rate at start of treatment (mm/hr), median (IQR)	69 (25-98)	84 (29.3-98.5)	0.841	27	0.796
Baseline AST (IU/L), median (IQR)	28 (14.8-41.8)	26 (17.8-38.8)	0.055	20	0.522
Baseline ALT (IU/L), median (IQR)	16 (10.3-27.8)	21.5 (11-40)	-0.079	20	0.469
Highest AST during therapy (IU/L), median (IQR)	27 (18-46)	27 (21-38.5)	-1.070	30	0.147
Highest ALT during therapy (IU/L), median (IQR)	16 (10-25)	25 (13.3-48)	-0.773	31	0.223

The most commonly identified *Staphylococcus* spp. was methicillin-susceptible *S. aureus* (MSSA), with an incidence rate of 50% in both groups (Table 3).

**Table 3:** Microbiology Characteristics *Multiple patients in the non-RIF group had multiple *Staphylococcus *spp. isolated. RIF: rifampin

Pathogens	RIF Combo (n=16), n (%)	Non-RIF* (n=18), n (%)
Methicillin-sensitive *Staphylococcus aureus* (MSSA)	8 (50)	9 (50)
Methicillin-resistant *Staphylococcus aureus* (MRSA)	3 (18.8)	2 (11.1)
Methicillin-sensitive coagulase-negative *Staphylococci* (CoNS)	3 (18.8)	4 (22.2)
Methicillin-resistant coagulase-negative *Staphylococci* (CoNS )	2 (12.5)	3 (16.7)

Vancomycin was the most frequently used antibiotic in both groups, with more than 87% of patients receiving this treatment initially, followed by de-escalation as appropriate once culture susceptibility results returned. Of note, some antimicrobials (i.e., ertapenem) were utilized in combination with others (i.e., cefazolin) to help sterilize the source or other potential metastatic sites. Tetracyclines were the predominant choice for suppressive therapy among patients. Antimicrobial treatment and suppression regimens are detailed in Table 4.

**Table 4:** Antimicrobial Therapies Utilized *cefadroxil, cephalexin, piperacillin/tazobactam

Antimicrobials	RIF Combo (n=16), n (%)	Non-RIF (n=18), n (%)
Initial treatment phase (first 6 weeks)		
Vancomycin	14 (87.5)	17 (94.4)
Daptomycin	4 (25)	2 (11.1)
Ceftaroline	0	1 (5.6)
Sulfamethoxazole-trimethoprim (SMX-TMP)	1 (6.3)	0
Doxycycline	0	3 (16.7)
Cefazolin	5 (31.3)	6 (33.3)
Oxacillin	5 (31.3)	4 (22.2)
Cefepime	1 (6.3)	10 (55.6)
Ertapenem	0	1 (5.6)
Another beta-lactam-based regimen*	1 (6.3)	3 (16.7)
Oral suppression treatments (after initial treatment)		
Cefadroxil	4 (25)	3 (16.7)
Cephalexin,	5 (31.3)	5 (27.8)
Doxycycline	5 (31.3)	10 (55.6)
Minocycline	3 (18.8)	1 (5.6)
Sulfamethoxazole-trimethoprim	1 (6.3)	0

The primary outcome of treatment failure (Table 5) was significantly different in the non-RIF group (50%) compared with the RIF group (12.5%) (P = 0.030). A total of two patients in the RIF group experienced treatment failure, while nine patients in the non-RIF group had treatment failure. In the RIF group, one patient had treatment failure within three months of the index surgery, and the other patient experienced treatment failure between three and six months. Among the non-RIF group, six patients had treatment failure within three months, one patient between three and six months, and two patients between six and 12 months from the index surgery. The reasons for treatment failure in the RIF group were a lack of infection eradication in one patient and reinfection with a new microorganism in one patient. In the non-RIF group, treatment failure was attributed to the need for subsequent surgery in seven out of nine patients, reinfection with a new microorganism in two patients, and lack of infection eradication in one patient.

Implants were removed at a non-significantly lower rate in the RIF group compared to the non-RIF group (18.8% vs. 38.9%, P = 0.270). Both groups utilized long-term suppressive therapy at a similar rate, with lifelong therapy being the most common duration. Hepatotoxicity was observed in the RIF group only, affecting two out of 16 patients. Rifampin was stopped in both of the affected patients with subsequent resolution of liver function test abnormalities. There were no reported instances of resistance to rifampin therapy for any isolated Staphylococcus spp., and rifampin was always used in combination with other antimicrobials. The median duration of rifampin therapy was 42 days, with a median total daily dose of 600 mg. Rifampin was discontinued prematurely in two patients due to gastrointestinal disturbances or transaminitis. Lastly, there was one reported case of PJI-related mortality within one year from the index surgery, which occurred in a patient in the RIF group (Table 5).

**Table 5:** Outcomes *All p-values were obtained with Fisher's Exact Test.

Outcomes	RIF Combination (n=16), n (%)	Non-RIF (n=18), n (%)	P-value*
Primary Outcome			
Total treatment failure	2 (12.5)	9 (50)	0.030
3-month treatment failure	1 (6.3)	6 (33.3)	0.090
3-6-month treatment failure	1 (6.3)	1 (5.6)	1
6-12-month treatment failure	0	2 (11.1)	0.487
Secondary Outcomes			
Implant removal	3 (18.8)	7 (38.9)	0.270
Long-term suppressive therapy utilized	13 (81.3)	14 (77.8)	1
Duration of long-term suppressive oral antibiotics			0.684
≤ 3 months	0	0	
3 to 6 months	1 (7.7)	2 (14.3)	
> 6 to 12 months	1 (7.7)	3 (21.4)	
> 12 months	1 (7.7)	0	
Lifelong (chronic)	10 (76.9)	9 (64.3)	
Hepatotoxicity	2 (12.5)	0	1
PJI-related mortality within 1 year	1 (6.3)	0	0.214

## Discussion

Rifampin's ability to penetrate biofilms makes it a promising option for treating PJI, despite conflicting evidence in the literature regarding its benefits [12]. Our study provides additional supporting evidence that rifampin use can significantly reduce treatment failure rates in staphylococcal PJI, similar to other reported studies [13,14].

IDSA guidelines recommend using rifampin for up to three to six months in patients with staphylococcal PJI who undergo surgical management with a DAIR strategy [1]. However, many patients are unable to tolerate this recommended duration due to ADEs or drug-drug interactions. Suzuki et al. demonstrated the benefits of rifampin treatment in *S. aureus* PJI for a period of up to 180 days following DAIR [14]. Notably, this benefit was observed even though the median duration of rifampin therapy was only 36 days. Furthermore, subgroup analyses showed a benefit primarily in knee PJI (45% reduced risk of recurrence from day one to 90 and 81% reduction from day 91 to 180) compared to hip PJI (no reduction in risk at any point from day one to 180). Similarly, Tai et al. found that rifampin use reduced treatment failure after DAIR primarily in knee PJI (95%CI, 0.20-0.79) (P = 0.008), as opposed to hip PJI (95%CI, 0.35-6.15) (P = 0.597) [15]. Another retrospective study, involving 669 cases, demonstrated a statistically significant difference in preventing treatment failure when comparing rifampin combination therapy to monotherapy (without rifampin) in patients with primarily *S. aureus* PJI. The study reported a treatment failure rate of 32.2% for combination therapy, whereas monotherapy had a rate of 54.2% (p<0.001). The effect was most notable for knee joint-associated PJI with a treatment failure rate of 28.6% for combination therapy and 63.9% for monotherapy, thus favoring rifampin addition [13]. In our study, the median duration of rifampin therapy was 42 days, and adjunctive rifampin was associated with a statistically significant decrease in treatment failure (P = 0.030). Our study's findings complement the existing literature.

Existing literature suggests that the use of antibiotic suppressive therapy following initial IV antimicrobial treatment can reduce the risk of treatment failure, particularly in cases involving DAIR. Tai et al. found that a shorter duration of oral antibiotics after IV treatment (90 days vs. one year) was associated with an increased risk of recurrent infection (95%CI, 1.48-8.25) (P = 0.005) [15]. Similarly, Shah et al. reported a significantly higher survival probability in patients who received greater than six weeks of oral suppressive therapy after initial IV therapy compared to those who only received the initial IV therapy (68% vs. 39.4%, P = 0.009) [16]. In our study, there was no difference in the utilization of long-term antibiotic suppressive therapy between the two groups. It is important to acknowledge, however, that our study did not collect data on dosing regimens or drug concentrations, which limits our ability to fully assess the impact of the IV and oral antibiotic regimens used.

Ultimately, the findings of this study demonstrate the benefits of using rifampin in the treatment of PJI at our institution. In clinical practice, risks and benefits should be assessed before incorporating rifampin into a PJI treatment regimen. The strengths of this study include its focus on *Staphylococcus* spp., which are the most common pathogens associated with PJI, as well as the inclusion of different surgical modalities such as DAIR and one-stage exchange procedures in the management of PJI. Limitations include a single-center retrospective design, the limited number of patients on rifampin combination therapy leading to a small overall sample size, the higher burden of diabetes in the non-rifampin group, not capturing of patients who received antimicrobial beads or topical antibiotics during surgery, and the exclusion of polymicrobial PJI. Of note, our study was not powered to stratify the results by type of surgery or joint involved. Additionally, treatment failure due to suboptimal antibiotic dosing was not assessed as serum drug concentrations were not collected. Further randomized controlled studies are needed to provide clarity on the impact of surgical treatment modality, antimicrobial dosing with or without TDM, long-term suppressive antibiotic therapy, and the influence of polymicrobial sources on PJI outcomes.

## Conclusions

Current literature on the use of rifampin in the treatment of PJI has yielded variable results. The results of our study demonstrate that the utilization of rifampin in staphylococcal PJI led to a statistically significant reduction in treatment failure at our institution. Our study, therefore, adds to the body of literature supporting the use of rifampin as adjunctive treatment for staphylococcal PJI.
